# Canine Circovirus Suppresses the Type I Interferon Response and Protein Expression but Promotes CPV-2 Replication

**DOI:** 10.3390/ijms23126382

**Published:** 2022-06-07

**Authors:** Xiangqi Hao, Yanchao Li, Hui Chen, Bo Chen, Ruohan Liu, Yidan Wu, Xiangyu Xiao, Pei Zhou, Shoujun Li

**Affiliations:** 1 College of Veterinary Medicine, South China Agricultural University, Guangzhou 510642, China; haoxiangqi0829@163.com (X.H.); liyanchao@stu.scau.edu.cn (Y.L.); chan.scau.edu.cn@stu.scau.edu.cn (H.C.); chenbovet@stu.scau.edu.cn (B.C.); liuruohan@stu.scau.edu.cn (R.L.); wuyidan@stu.scau.edu.cn (Y.W.); 13030158655@163.com (X.X.); 2 Guangdong Provincial Key Laboratory of Prevention and Control for Severe Clinical Animal Diseases, Guangzhou 510642, China; 3 Guangdong Provincial Pet Engineering Technology Research Center, Guangzhou 510642, China

**Keywords:** canine circovirus, coinfection, interferon response, protein expression inhibition, canine parvovirus

## Abstract

Canine circovirus (CanineCV) is an emerging virus in canines. Since the first strain of CanineCV was reported in 2012, CanineCV infection has shown a trend toward becoming a global epidemic. CanineCV infection often occurs with coinfection with other pathogens that may aggravate the symptoms of disease in affected dogs. Currently, CanineCV has not been successfully isolated by laboratories, resulting in a lack of clarity regarding its physicochemical properties, replication process, and pathogenic characteristics. To address this knowledge gap, the following results were obtained in this study. First, a CanineCV strain was rescued in F81 cells using infectious clone plasmids. Second, the Rep protein produced by the viral packaging rescue process was found to be associated with cytopathic effects. Additionally, the Rep protein and CanineCV inhibited the activation of the type I interferon (IFN-I) promoter, blocking subsequent expression of interferon-stimulated genes (ISGs). Furthermore, Rep was found to broadly inhibit host protein expression. We speculate that in CanineCV and canine parvovirus type 2 (CPV-2) coinfection cases, CanineCV promotes CPV-2 replication by inducing immunosuppression, which may increase the severity of clinical symptoms.

## 1. Introduction

Circoviruses are non-enveloped, single-stranded circular DNA viruses with an icosahedral symmetrical structure. Canine circovirus (CanineCV) is a newly discovered mammalian circovirus that was first reported by Kapoor et al. in 2012 [1]. Subsequently, CanineCV was identified in Italy and was thought to be the causative agent of canine necrotizing vasculitis and lymph node granuloma, which can cause vomiting and hemorrhagic diarrhea in dogs [2,3]. To date, CanineCV has been reported in several countries, indicating that the virus has spread worldwide [4,5,6,7,8,9,10,11,12]. The genome of CanineCV is approximately 2063–2064 nt in size and primarily contains three open reading frames (ORFs). ORF1 encodes the replicase protein (Rep), ORF2 encodes the capsid protein (Cap), and the ORF3-encoded protein is less studied. Dogs of all ages and both sexes can be infected with CanineCV, and the prevalence of CanineCV infection is high [2,10,13,14,15,16]. In addition to dogs, CanineCV has been detected in a variety of wild animals and even domestic cats and cattle [5,17,18,19], suggesting that the virus has a large host range and that studying its pathogenic mechanisms is essential.

To date, no researchers have successfully isolated CanineCV because it is difficult to obtain samples infected with CanineCV alone. Notably, researchers have constructed double gene copy infectious clones of porcine circovirus (PCV) and successfully expressed them in PK-15 cells, which has promoted progress in circovirus research [20].

Studies have shown that some circoviruses affect the host interferon response [21]. The innate immune response is the first line of defense of a host. During the response to different viral infections, type I interferon (IFN-I) can be produced in most cells [22]. Activation of IFN-I is mediated primarily by the interferon regulatory factor 3 (IRF3), nuclear factor kappa-B (NFκB), and nuclear transcription activator protein-1 (AP1) signaling pathways. Activated IFN-I can also stimulate the activation of interferon-stimulated response elements (ISREs) that promote the expression of interferon-stimulated genes (ISGs), leading to antiviral effects. Previous studies have revealed that CanineCV not only is present in the intestine but also infects the spleen and lymphoid tissue, replicating in macrophages and potentially inducing immunosuppression [3]. Therefore, it is important to explore the effects of CanineCV on the host immune system. CanineCV is frequently detected with other viruses in fecal samples from dogs with diarrhea, and when canine parvovirus type 2 (CPV-2) coinfection is present, the mortality rate increases from 22.2% (monoinfection with CPV-2) to 43.8% [10]. This pattern suggests that CanineCV coinfection may affect the host antiviral response, leading to severe clinical symptoms in sick dogs.

In this study, CanineCV was rescued in Feline kidney (F81) cells with infectious clones, and the mechanism by which CanineCV blocks the IFN-I response and protein expression was described. More importantly, the Rep protein expression increased the replication level of CPV-2. In summary, the mechanism by which CanineCV promotes the replication of CPV-2 by inducing immunosuppression, which may increase the severity of clinical symptoms, is revealed.

## 2. Results

### 2.1. CanineCV Rescue

#### 2.1.1. Construction of Infectious Clones of CanineCV

**Figure 1:**
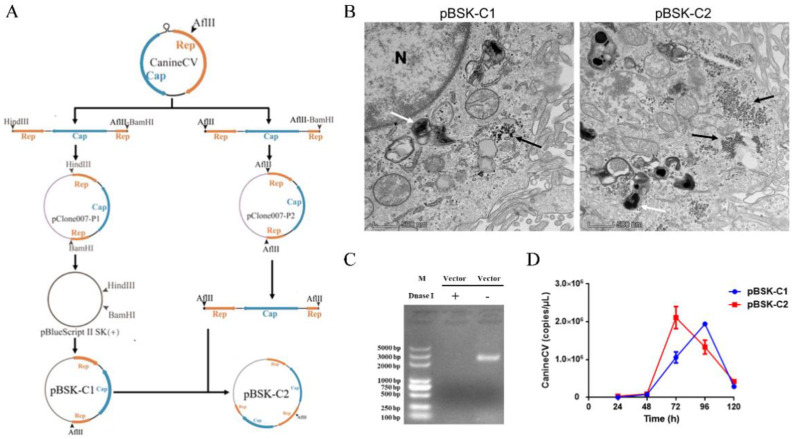
CanineCV rescue. (**A**) Schematic diagram of the steps for constructing the CanineCV single gene copy and double gene copy infectious clone plasmids. pClone007-P1 and pClone007-P2 were constructed as intermediate plasmids. Eventually, pBSK-C1 and pBSK-C2 were used for virus rescue. (**B**) TEM images of F81 cells transfected with pBSK-C1 and pBSK-C2. F81 cells were inoculated into 6-well plates, and 2 µg of plasmid was transfected into the cells. Seventy-two hours after transfection, cells were collected and processed for observation. CanineCV particles are labeled with black arrows. Inclusion bodies are labeled using white arrows. TEM showed that pBSK-C2-transfected cells exhibited greater viral packaging. N, cell nucleus. The scale bar represents 500 nm. (**C**) The pBlueScript II SK (+) vector (2 µg) was digested according to the instructions, and electrophoresis was then performed to demonstrate the proper activity of DNase I. (**D**) qPCR was used to determine the number of rescued CanineCV particles at different time points, and both experimental groups showed the trend of an initial increase followed by a decrease in the number of virions.

First, primers were designed to construct the intermediate plasmids pClone007-P1 containing a single gene copy and pClone007-P2 containing two gene copies. Then, the process shown in Figure 1A was followed to construct the corresponding virus-rescued infectious clone plasmids pBSK-C1 and pBSK-C2.

#### 2.1.2. Ultrastructural Characteristics of CanineCV-Infected Cells

pBSK-C1 and pBSK-C2 were transfected into F81 cells. Seventy-two hours after transfection, cells were collected, fixed, sectioned, stained, and observed by TEM. Numerous viral particles of approximately 20 nm in diameter were observed in the cytoplasm of the infected cells, as were numerous inclusions (Figure 1B). In summary, CanineCV was successfully rescued, and pBSK-C2-transfected cells exhibited greater viral packaging than pBSK-C1-transfected cells.

#### 2.1.3. Growth Characteristics of Rescued Viruses in Cells

pBSK-C1 and pBSK-C2 were transfected into F81 cells using Lipo8000, and the viral copy number was determined at 24 h, 48 h, 72 h, 96 h, and 120 h after transfection. Cells were collected, and DNase I was added to eliminate residual plasmids, with an equal amount of plasmid used as the control to ensure the proper activity of DNase I (Figure 1C). Then, viral DNA was extracted and analyzed by qPCR. The number of virions in pBSK-C1- and pBSK-C2-transfected cells increased gradually, and the number of virions in the pBSK-C1 group peaked at 96 h and decreased thereafter to 120 h (Figure 1D). The number of virions in the pBSK-C2-transfected group peaked at approximately 72 h and then decreased gradually to 96 h (Figure 1D). In brief, CanineCV was successfully rescued via both strategies, and pBSK-C2-transfected cells exhibited packaging of more virions in less time. Unfortunately, the rescued CanineCV could be blindly transmitted to F81 cells for only four generations, after which the virus gradually disappeared (data not shown).

### 2.2. CanineCV and Rep Affect Cell Viability

#### 2.2.1. CanineCV Affects Cell Viability

**Figure 2:**
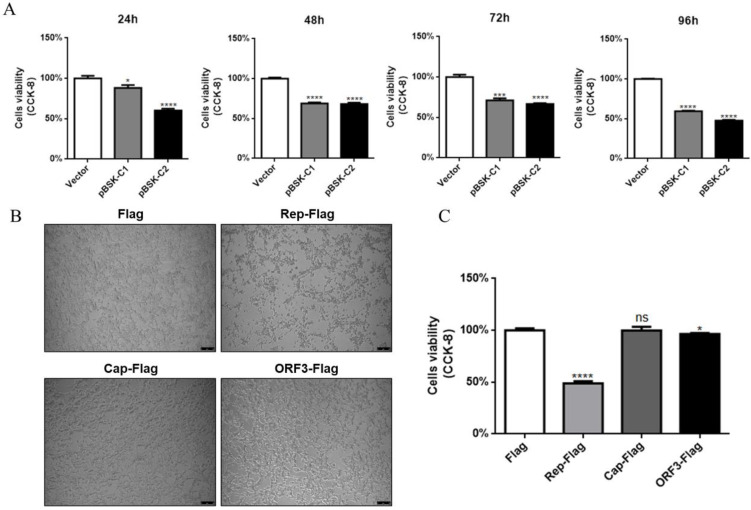
CanineCV and Rep affected cell viability. (**A**) HEK-293T cells were transfected with empty vector, pBSK-C1, or pBSK-C2; CCK-8 reagent was then added at 24 h, 48 h, 72 h, and 96 h; and the OD450 value was measured after incubation. The production of CanineCV consistently affected cell viability. (**B**) HEK-293T cells were transfected with different viral expression plasmids. The growth status of each group of cells was directly imaged under an optical microscope, and Rep-protein-expressing cells showed CPEs. (**C**) A CCK-8 assay was performed to detect the effect of each viral protein on cell viability at 48 h. The cell viability in the Rep protein expression group was significantly lower than that in the other groups. The data shown in the figure are presented as the mean ± SD of three independent experiments. * *p* < 0.05, *** *p* < 0.001, **** *p* < 0.0001 and ns means no significance compared with the empty vector transfection group.

To investigate the effect of CanineCV on cell viability, a CCK-8 assay was used to determine cell viability after transfection with pBSK-C1 and pBSK-C2. The reagent was added to cells and incubated for 24 h, 48 h, 72 h, and 96 h. The OD450 value was measured after incubation at 37 °C for 30 min. Both the single gene copy and double gene copy infectious clones affected cell viability and exhibited toxicity. Unfortunately, pBSK-C2 had a more severe effect on cells, suggesting that CanineCV produces proteins that are harmful to cells (Figure 2A).

#### 2.2.2. The Rep Protein Affects Cell Viability

We assumed that CanineCV encodes proteins that affect normal biological functions in cells during its replication. To identify the protein that affects cell viability, the three largest ORFs in CanineCV were selected and inserted into expression plasmids with 3×Flag and EGFP tags. Interestingly, the Rep protein affected cell morphology, and infected cells exhibited severe lesions, such as shrinkage and shedding (Figure 2B). Furthermore, the cell viability in the Rep protein expression group was significantly lower than that in the control group; however, Cap and ORF3 proteins exerted less obvious effects on cell viability than did Rep (Figure 2C). In conclusion, among the tested CanineCV-encoded proteins, the expression of Rep has the greatest impact on cell viability.

### 2.3. CanineCV and Rep Inhibit IFN-I and ISG Expression

**Figure 3:**
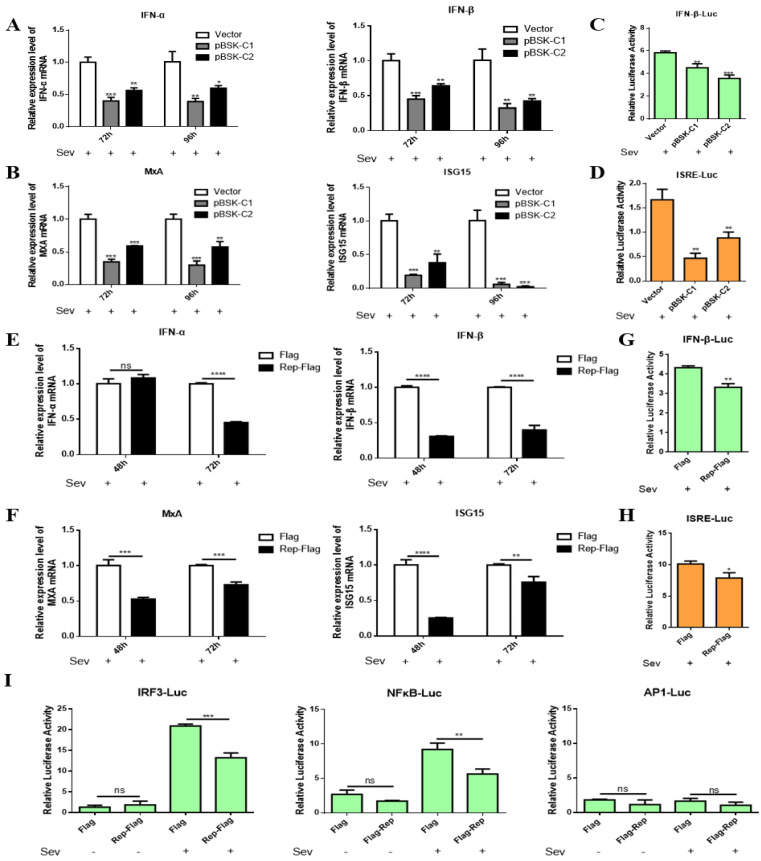
CanineCV and Rep inhibit IFN-I and ISG expression. F81 cells were transfected with different plasmids, total RNA was extracted from the cells at the indicated time points, and the mRNA levels of IFN-α, IFN-β (**A**,**E**), MxA and ISG15 (**B**,**F**) were measured by qPCR. In the dual luciferase assay, F81 cells were transfected with different plasmids for 24 h, inoculated with SeV and incubated for another 12 h, and lysed for determination of luciferase activity (**C**,**D**,**G**–**I**). CanineCV and the Rep protein inhibited SeV-activated IFN-α, IFN-β, MxA, and ISG15 expression at the mRNA level. Second, the results of the dual luciferase assay showed that CanineCV and the Rep protein blocked the activation of the IFN-β and ISRE promoters. Finally, the Rep protein blocked the interferon signaling pathway through the IRF3 and NFκB pathways. * *p* < 0.05, ** *p* < 0.01, *** *p* < 0.001, **** *p* < 0.0001 compared with the empty vector transfection group.

Previous studies have shown that CanineCV replicates mainly in macrophages and monocytes in lymphoid tissue and may induce immunosuppression [3]. To determine whether CanineCV inhibits the IFN-I response, F81 cells were transfected with pBSK-C1 and pBSK-C2. Prior to cell collection, the cells were inoculated with Sendai virus (SeV) and incubated for 12 h to stimulate the cellular IFN-I response, and then mRNA levels were measured by qPCR. The mRNA levels of IFN-α/β and ISGs were significantly decreased (Figure 3A,B). These results suggest that CanineCV inhibits IFN-I production in F81 cells. To further confirm that CanineCV inhibits the IFN-I response, F81 cells were cotransfected with IFNβ-Luc, pRL-TK and pBSK-C1 or pBSK-C2. Twenty-four hours post-transfection, the cells were inoculated with SeV and incubated for another 12 h. Finally, luciferase activity was measured. As expected, SeV-stimulated IFN-β promoter activity was significantly reduced upon infection with CanineCV (Figure 3C). These results indicate that CanineCV indeed inhibits the IFN-I response. Similarly, the activation of ISREs was inhibited (Figure 3D), indicating that the antiviral response was blocked.

Since the Rep protein severely affected cell viability, we hypothesized that it plays a major role in the regulation of IFN-I. To test this hypothesis, F81 cells were transfected with a plasmid encoding Rep, and IFN-I and ISG mRNA levels were then measured. The mRNA level of IFN-I was reduced (Figure 3E,F). Similarly, IFN-β promoter and ISRE activity were significantly decreased because of Rep protein expression (Figure 3G,H). The above results suggest that the Rep protein inhibits the IFN-I response.

Activation of IFN-β gene expression requires the involvement of multiple transcription factors, such as IRF3, NF-kB, and AP-1. To determine whether the activation of some of these transcription factors is inhibited, plasmids carrying different transcription factor sequences were transfected into F81 cells along with p3×Flag-Rep. Luciferase activity was measured, and Rep was found to block the activation of IRF3 and NF-kB but not AP-1 (Figure 3I). These data suggest that Rep inhibits IFN-β promoter activation by blocking the activation of NF-kB and IRF3. Thus, it was concluded that the Rep protein can block the IFN-I response through both the IRF3 and NF-kB pathways, which in turn affects antiviral gene transcription.

### 2.4. CanineCV and Rep Affect Protein Expression

**Figure 4:**
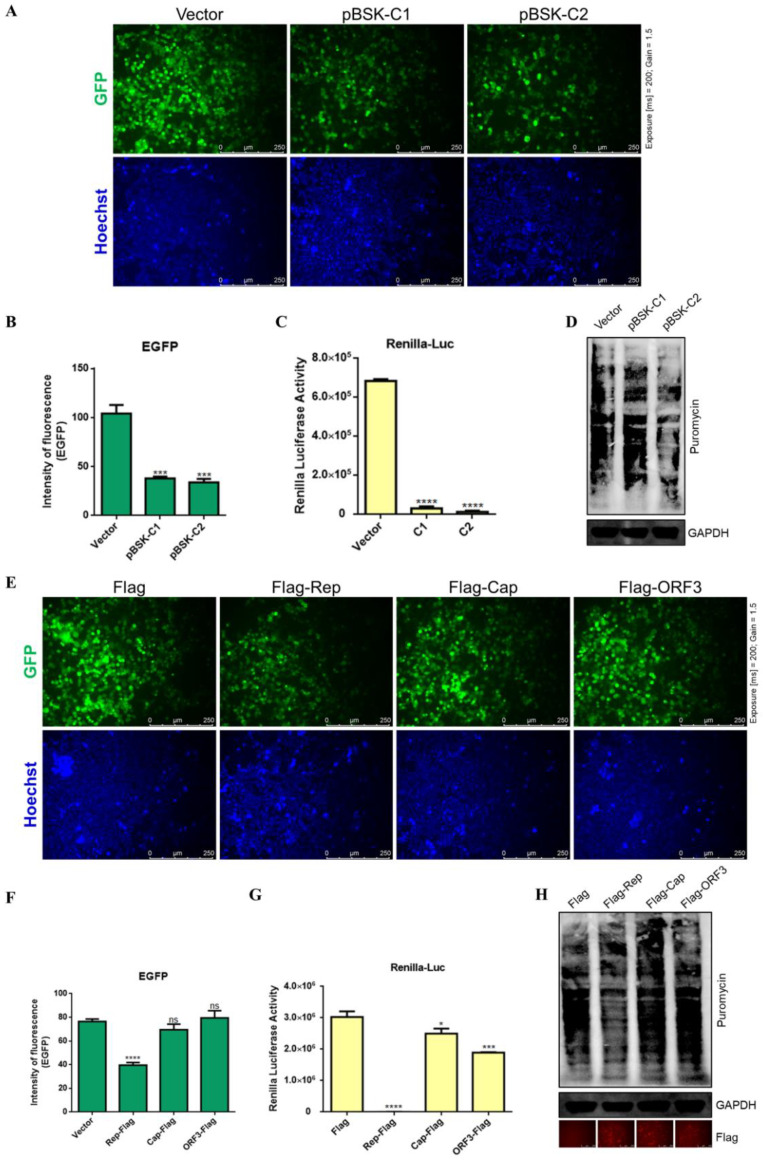
CanineCV and Rep inhibited protein expression. (**A**,**E**) F81 cells were cotransfected with EGFP-tagged protein expression plasmids and different viral plasmids. After 48 h, the cells were imaged under a fluorescence microscope (Exposure [ms] = 200; Gain = 1.5), and the fluorescence intensity, indicating the level of protein expression, was calculated using ImageJ (**B**,**F**). The results suggest that CanineCV and the Rep protein affected the expression of EGFP. (**C**,**G**) F81 cells were cotransfected with the pRL-TK plasmid and different viral plasmids. After 48 h, the cells were collected and Renilla luciferase activity was measured. Luciferase activity indirectly reflects the level of protein expression in cells. (**D**,**H**) Forty-eight hours after transfection, puromycin (10 μg/mL) was added to the medium and incubated at 37 °C for 30 min. The cells were washed twice with PBS and then lysed for WB analysis. Nascent peptides labeled with puromycin were detected with an anti-puromycin monoclonal antibody. An anti-Flag antibody was used to confirm viral protein expression (red), and an anti-GAPDH antibody was used as the internal reference. The WB analysis results showed that in the CanineCV rescue group and the Rep protein expression group, fewer peptides were labeled by puromycin, and cellular translation levels were low. * *p* < 0.05, *** *p* < 0.001, **** *p* < 0.0001.

Inhibition of cellular protein expression after viral infection is an important pathogenic mechanism. Viruses are dependent on host cells for replication, and they usually hijack the host translation system and evade intrinsic immunity [23]. To determine the effect of CanineCV on protein expression, the pEGFP-C3 vector was cotransfected with pBSK-C1 or pBSK-C2 into HEK-293T cells. After 48 h, EGFP expression was assessed. In addition, live cells were stained using Hoechst 33342 to determine cell numbers. All fields of view were randomly imaged at the same excitation intensity (Exposure [ms] = 200; Gain = 1.5). The expression levels of EGFP in cells infected with CanineCV were lower (Figure 4A,B). Renilla luciferase is often used as an internal reference fluorophore in dual luciferase assays because its protein expression is stable and can be measured by instrumentation. To further determine whether CanineCV affects protein expression, the pRL-TK plasmid was cotransfected with pBSK-C1 or pBSK-C2 into HEK-293T cells. Forty-eight hours later, Renilla luciferase activity was very low in the CanineCV group (Figure 4C). In addition, cellular peptide synthesis was monitored to determine whether CanineCV affects translation. Puromycin was added to label nascent peptides so that the translation efficiency of the cells was monitored. These experimental methods were described in previous studies [24,25]. As shown in Figure 4D, host translation was terminated during viral packaging. Specifically, the pBSK-C2-transfected group had the lowest protein translation level. These results indicate that CanineCV greatly affects the expression of host proteins.

To investigate the effects of viral proteins on protein expression, EGFP-tagged protein expression plasmids and plasmids containing the three ORFs were cotransfected into HEK-293T cells, and after 48 h, the expression of EGFP was assessed. Notably, the Rep protein significantly inhibited the expression of EGFP (Figure 4E,F). Moreover, these results were consistent with those of the Renilla luciferase assay; the Cap protein and ORF3 protein also slightly inhibited protein expression but to a much lesser degree than did the Rep protein (Figure 4G). The Cap protein and ORF3 protein also slightly inhibited protein translation, indicating that the method of measuring Renilla luciferase activity is more sensitive than the method of microscopic observation of EGFP. The WB analysis results showed decreased synthesis of puromycin-tagged nascent proteins and low cellular translation levels during Rep protein expression (Figure 4H). These results suggest that the Rep protein plays a major role in suppressing cellular protein expression.

### 2.5. CanineCV and the Rep Protein Promote CPV-2 Replication

**Figure 5:**
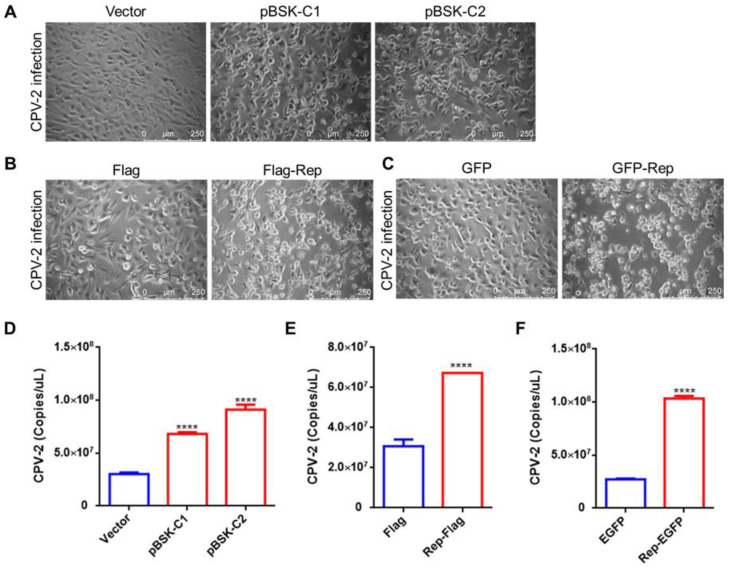
CanineCV and Rep promoted CPV-2 replication. F81 cells were transfected with the pBSK, pBSK-C1, or pBSK-C2 plasmid. Twenty-four hours later, the cells were inoculated with CPV-2 at an MOI of 1. After 24 h of infection, the CanineCV rescue group developed severe CPEs (**A**). Then, cells were harvested 48 h after viral infection, DNA was extracted, and qPCR was used to determine the CPV-2 copy number. The results suggested that the CPV-2 copy number in the CanineCV group was 2–3-fold higher than that in the control group (**D**). F81 cells were transfected with empty vector or the Rep plasmid. Twenty-four hours later, the cells were inoculated with CPV-2 at an MOI of 1. The Rep protein expression group showed significant cell lesions (**B**,**C**) and higher levels (2–3-fold) of CPV-2 replication (**E**,**F**). The data shown in the figure are presented as the mean ± SD of three independent experiments. **** *p* < 0.0001 compared with the empty vector transfection group.

Previous studies have indicated that most CanineCV-positive dogs with diarrhea are infected with more than one pathogen, with the highest proportion exhibiting coinfection with CPV-2 and CanineCV. CanineCV may act as a cofactor in the process of CPV-2 infection [7,10]. In this study, to investigate whether CanineCV infection promotes CPV-2 replication, F81 cells were transfected with pBSK-C1 and pBSK-C2 and were then inoculated with CPV-2 24 h later. Compared to control cells and cells in the CanineCV and Rep groups, cells quickly (24 h) showed cytopathic effects (CPEs) (Figure 5A–C). After CPV-2 infection for 48 h, total DNA was extracted and CPV-2 replication was assessed by qPCR. The results suggested that CanineCV infection increases the replication level of CPV-2 by 2–3-fold (Figure 5D). More interestingly, transfection of Flag-Rep or EGFP-Rep was also sufficient to promote CPV-2 replication in cells (Figure 5E,F).

## 3. Discussion

CanineCV is thought to be related to canine hemorrhagic diarrhea, vasculitis, and lymph node granuloma [2,3,4]. To date, few studies have examined the pathogenicity of CanineCV. In this study, infectious clones of CanineCV were constructed, and the virus was rescued in F81 cells. Studying the effects of CanineCV on innate immunity and cell viability provides the basis for animal experiments on CanineCV and helps to guide studies on CanineCV pathogenicity and coinfection with other pathogens in dogs.

Although the virus is prevalent in several regions, to date, there are no reports of successful isolation of CanineCV. Some scholars have attempted to culture CanineCV using various cell lines, but all attempts failed [2,19]. In this research, infectious clones of CanineCV with one and two gene copies were constructed, and the virus was rescued in F81 cells. F81 cells were selected because they have higher transfection efficiency than Madin–Darby canine kidney cells. Numerous CanineCV virus particles in the cytoplasm of infected cells were observed by TEM; these particles had a diameter of approximately 20 nm, consistent with the typical size of circoviruses [26]. Inclusion bodies appeared in infected cells, with different sizes, shapes, and electron densities, similar to previous results observed in canine tissues and cells [3]. The results of this study suggest that double gene copy infectious clones produce more virions than single gene copy clones. Unfortunately, similar to the findings of other researchers [2], the virus gradually disappeared after four generations of blind transmission in F81 cells (data not shown), and the reason requires further study.

Viruses often evolve multiple mechanisms to escape host immune defenses. In the present study, we found that CanineCV and its encoded Rep protein severely affect cell viability. Furthermore, activation of the IFN-β promoter was inhibited, and IFN-α and IFN-β mRNA expression was inhibited during CanineCV infection or Rep protein expression. The viral Rep protein inhibited the activation of the IFN-β promoter mainly through two signaling pathways, IRF3 and NFκB; as a result, ISRE activation was inhibited, and the production of antiviral proteins was impeded. In addition, CanineCV and Rep suppress protein expression, another very important pathogenic mechanism. Overall, this study provides evidence that CanineCV induces immunosuppression.

Several recent studies have shown that coinfection of CanineCV with CPV-2 leads to increased mortality in dogs [7,15,27], and we hypothesized that this effect is related to CanineCV-induced immunosuppression. Our study showed that CanineCV infection or Rep protein expression inhibits the IFN response yet facilitates CPV-2 replication in cells. Therefore, dogs infected with CanineCV may exhibit more severe clinical signs when coinfected with other viruses. It is worth noting that the probability of coinfection of CPV-2, canine distemper virus (CDV), and canine coronavirus (CCoV) with CanineCV reached 100% [19,28], 70–80% [27,29,30], and 50% [7,31], respectively. Thus, it is clear that, apart from DNA viruses, CDV and CCoV among RNA viruses are also coinfected with CanineCV, but the coinfection of CanineCV with CPV-2 is more common in dogs. Similar results were reported in other animal circovirus infections. For example, pigs coinfected with PCV2 and porcine parvovirus (PPV) became dull, developed jaundice and hepatomegaly, and some even died [32]. Coinfection of PCV2 or PCV3 with PPV leads to severe viremia and reproductive disorders [33,34,35]. In addition, PCV-2 infection inhibits the response of immune-related signaling pathways and promotes the replication of viruses such as pseudorabies virus and porcine reproductive and respiratory syndrome virus [36,37]. Worse still, duck circovirus also causes immunosuppression, and coinfection with other pathogens can lead to serious consequences [38,39].

CanineCV will need to be given additional attention because it is gradually showing the ability to spread across species. CanineCV has a high prevalence in wild animals such as foxes, wolves, and badgers. More concerningly, CanineCV has been found in samples from cows and domestic cats in northeastern China [17]. CanineCV induces immunosuppression to promote the replication of other viruses, increasing disease severity, which imposes a further burden on public health safety.

## 4. Materials and Methods

### 4.1. Cells, Viruses, and Plasmids

F81 cells and HEK-293T cells were cultured in Dulbecco’s modified Eagle’s medium (DMEM) supplemented with 10% fetal bovine serum (FBS) in 5% CO_2_ at 37 °C. The CanineCV-DG strain was obtained from previous studies [40]. CPV-2 and SeV were stored in our laboratory. The pClone007 vector was purchased from Tsingke Biotechnology Co., Ltd. The empty p3×Flag-CMV10 and pEGFP-C3 vectors and the pBlueScript II SK (+) vector were purchased from Wuhan Miaoling Biotechnology (Wuhan, China). The pMD18-T clone vector was purchased from Takara Biomedical Co., Ltd. Technology (Beijing, China). The main ORFs of the virus were cloned and then ligated into the p3×Flag-CMV10 and pEGFP-C3 vectors. The feline interferon-associated luciferase reporter plasmids (IFN-β-Luc, NFκB-Luc, IRF3-Luc, and AP1-Luc) have been described in previous studies [41,42].

### 4.2. qPCR Analysis

For virus quantification, a pair of qPCR primers based on the Rep gene was designed as follows: q-Rep217F (5′-GCATAGTATTACCCGGCA-3′) and q-Rep217R (5′-GCATAGTATTACCCGGCA-3′). After amplification, amplicons were recovered from the positive bands and inserted into pMD18-T. After plasmid extraction, the standard plasmids were subjected to gradient dilution (10^9^~10^1^ copies/μL), and these solutions were ultimately used to establish the qPCR standard curve. To determine the number of CPV-2 virions in cells, qPCR was used according to a previous study [43].

To detect changes in the expression of interferon-related genes in cells, total RNA was extracted from cells and then reverse-transcribed into cDNA. The mRNA expression levels of different genes (IFN-α, IFN-β, ISG15, and MxA) were determined, and GAPDH was used as the housekeeping gene. The primers used are listed in Table A1. The relative mRNA expression of each target gene was calculated using the 2^−ΔΔCt^ method.

### 4.3. Construction of an Infectious Clone of CanineCV

The primers HindIII-F1 (5′-CACAAGCTTAGGACCTGCCGTATGGGTG-3′) and AflII-BamHI-R (5′-CGGGATCCCTTAAGGTTAACGAACCCTTGAAGG-3′) were first used for amplification to construct the single gene copy clone pClone007-P1. The primers AflII-F2 (5′-CATCTTAAGAGGACCTGCCGTATGGGTG-3′) and AflII-BamHI-R were used to construct the double gene copy infectious clone pClone007-P2. Next, the two intermediate plasmids mentioned above were modified. The pClone007-P1 and pBlueScript II SK (+) vectors were digested using the restriction endonucleases HindIII-HF and BamHI-HF, respectively, and the target fragments were purified and ligated to obtain a single gene copy clone for virus rescue, which was named pBSK-C1. A double gene copy infectious clone, pBSK-C2, was obtained by processing pBSK-C1 and pClone007-P2 with a similar method as described above.

### 4.4. Transmission Electron Microscopy (TEM)

F81 cells were seeded into a six-well cell culture plate and transfected with pBSK-C1 and pBSK-C2 using Lipo8000™ (Beyotime, Shanghai, China). Cells were cultured for 72 h and then collected and prepared for electron microscopy. Cells were collected by centrifugation and fixed, and the fixed cell masses were subjected sequentially to the following steps: PBS rinsing, 1% osmium fixation, ultrapure water rinsing, ethanol gradient dehydration, excessive acetone removal, gradient infiltration of an embedding agent, curing, block repair, sectioning, and staining. Finally, the dried sections were observed under a Talos L120C TEM.

### 4.5. Indirect Immunofluorescence Assay

F81 cells were inoculated into 24-well plates and transfected with p3×Flag, p3×Flag-Rep, p3×Flag-Cap, or p3×Flag-ORF3. After 24 h, the medium was discarded, and the cells were fixed with 4% paraformaldehyde for 20 min. Then, the cell membrane was permeabilized using 0.2% Triton X-100, and the cells were blocked in 5% skim milk and incubated with a mouse monoclonal ANTI-FLAG^®^ M2 antibody (Sigma-Aldrich, Taufkirchen, Germany) overnight at 4 °C. The primary antibody was discarded, and the cells were subsequently incubated overnight with goat anti-mouse IgG H&L (Alexa Fluor^®^ 594). Finally, an anti-fluorescence quenching tablet (including DAPI) (Solarbio, Beijing, China) was added to facilitate microscopic observation.

### 4.6. Cell Viability Assay

HEK-293T cells were seeded into 96-well plates, and the appropriate plasmid was transfected into the cells. CCK-8 reagent (Wanleibio, Shenyang, China) was added at different time points, and the absorbance (OD450 nm) was measured with a Thermo Scientific microplate reader after incubation at 37 °C for 1 h in a dark incubator.

### 4.7. Luciferase Assay

To analyze the effect of CanineCV and viral proteins on the IFN-β response, F81 cells (5×10^4^ cells/well) were cotransfected with 0.5 μg of the reporter plasmid and 0.02 μg of the pRL-TK plasmid (Promega). The cells were also transfected with CanineCV infectious clone plasmids or viral protein expression plasmids 24 h later. After transfection for 24 h, the cells were inoculated with SeV (100 hemagglutinating activity units/well). After 12 h of SeV stimulation, the firefly and Renilla luciferase activities were measured. The relative luciferase activity (RLA) in each sample was calculated as the ratio of firefly luciferase activity to Renilla luciferase activity.

To evaluate the effect of CanineCV and its encoded proteins on protein expression, the pRL-TK plasmid and CanineCV infectious clone plasmids or viral protein expression plasmids were cotransfected into F81 cells. After 24 h, Renilla luciferase activity was measured.

### 4.8. Western Blot (WB) Analysis

Cells were washed three times with precooled PBS, lysed in protein lysis solution (Wanleibio, Shenyang, China) containing 1% protease inhibitor (Wanleibio, Shenyang, China), subjected to sodium dodecyl sulfate–polyacrylamide gel electrophoresis (SDS–PAGE), and finally transferred to polyvinylidene fluoride (PVDF) membranes (Millipore, Burlington, MA, USA). Membranes were blocked with 5% skim milk powder in Tris-buffered saline containing Tween 20 and incubated with an anti-GAPDH antibody (Bioss, Beijing, China) or an anti-puromycin antibody (Millipore, Burlington, MA, USA). Goat anti-mouse IgG H&L (IRDye^®^ 800 CW) was selected as the secondary antibody, and membranes were incubated with this antibody at room temperature for 30 min.

### 4.9. Statistical Analysis

In this study, Primer Premier 6.0 software was used for primer design. GraphPad Prism 8.0.1 was used for statistical analysis and graphing of the data.

## 5. Conclusions

**Figure 6:**
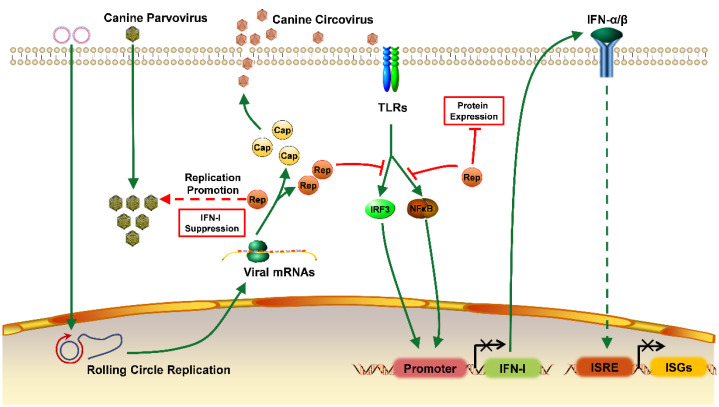
Schematic diagram of the effect of CanineCV on the IFN-I signaling pathway and CPV-2 replication. Infectious clones were transfected into cells, after which viral particles were packaged depending on the characteristics of the rolling loop replication. Then, viral protein expression was initiated, the host cell IFN-I immune pathway was blocked, protein synthesis was reduced, and antiviral defenses were dysregulated. CPV-2 replicates at higher levels in intracellular environments whereas antiviral genes are expressed at low levels.

In this study, CanineCV infectious clones were designed, and viral packaging and replication were evaluated using TEM and qPCR. CanineCV is localized mostly in the cytoplasm, with packaging titers peaking between 72 and 96 h. Interestingly, CanineCV suppresses the expression of IFN-I and ISGs and inhibits the expression of proteins. However, this mechanism facilitates CPV-2 replication (Figure 6). Therefore, coinfection with CPV-2 and CanineCV may result in more severe clinical symptoms than infection with CPV-2 alone.

## Data Availability

All data generated or analyzed during this study are included in this manuscript.

## Appendix A

**Table A1:** Primers used for RT-qPCR analysis.

Gene	Primers	Sequences (5′→3′)
IFN-α	IFN-α-F	GCCCTCTTCCTTCTTGGT
	IFN-α-R	GCCTTGTGGGACTGGTCT
IFN-β	IFN-β-F	GTGTGTTTCTTCACCACCGC
	IFN-β-F	GTGTCGCAAGGAGGTTCTCA
ISG15	ISG15-F	TCCTGGTGAGGAACCACAAGGG
	ISG15-R	TTCAGCCAGAACAGGTCGTC
MxA	MxA-F	TCAAGGGCGGAGATGGTT
	MxA-F	AAGGGAGTCGATGAGGTCAA
GAPDH	GAPDH-F	GTGATGCTGGTGCTGAGTATGT
	GAPDH-R	ATGAGTCCCTCCACGATGC

F, forward primer. R, reverse primer.
